# White matter microstructure organization across the transition to fatherhood

**DOI:** 10.1016/j.dcn.2024.101374

**Published:** 2024-04-12

**Authors:** Sofia I. Cárdenas, Yael Waizman, Van Truong, Pia Sellery, Sarah A. Stoycos, Fang-Cheng Yeh, Vidya Rajagopalan, Darby E. Saxbe

**Affiliations:** a Department of Psychology, University of Southern California, USA; b Department of Pediatrics, Children’s Hospital Los Angeles, Keck School of Medicine, University of Southern California, USA; c Department of Psychiatry and the Behavioral Sciences, Keck School of Medicine, University of Southern California, USA; d Department of Neurological Surgery, University of Pittsburgh, USA

**Keywords:** Transition to parenthood, Fathers, Diffusion-weighted imaging, Depression, Stress

## Abstract

The transition to parenthood remains an understudied window of potential neuroplasticity in the adult brain. White matter microstructural (WMM) organization, which reflects structural connectivity in the brain, has shown plasticity across the lifespan. No studies have examined how WMM organization changes from the prenatal to postpartum period in men becoming fathers. This study investigates WMM organization in men transitioning to first-time fatherhood. We performed diffusion-weighted imaging to identify differences in WMM organization, as indexed by fractional anisotropy (FA). We also investigated whether FA changes were associated with fathers’ postpartum mental health. Associations between mental health and WMM organization have not been rarely examined in parents, who may be vulnerable to mental health problems. Fathers exhibited reduced FA at the whole-brain level, especially in the cingulum, a tract associated with emotional regulation. Fathers also displayed reduced FA in the corpus callosum, especially in the forceps minor, which is implicated in cognitive functioning. Postpartum depressive symptoms were linked with increases and decreases in FA, but FA was not correlated with perceived or parenting stress. Findings provide novel insight into fathers’ WMM organization during the transition to parenthood and suggest postpartum depression may be linked with fathers’ neuroplasticity during the transition to parenthood.

## 1 Introduction

Adult neuroplasticity during the transition to parenthood is an underexplored area of research. New parents appear to experience neurobiological remodeling, as indicated by studies finding gray matter volume changes (Feldman, 2015, Kim et al., 2014, Paternina-Die et al., 2020). However, to our knowledge, longitudinal changes in white matter microstructural (WMM) organization have not yet been prospectively examined in human mothers or fathers transitioning to parenthood. Further, given prior evidence that WMM organization is associated with maternal mental health during the transition to parenthood, examining fathers' WMM organization in conjunction with stress and depression may yield insight into how the brain reflects fathers' adjustment to parenthood. The proposed study addresses the aforementioned gaps by integrating neuroimaging and psychological data to examine changes in fathers’ WMM organization across the transition to parenthood.

Extant research suggests that white matter changes across the lifespan. White matter makes up more than 50% of the brain and comprises a network of neural fibers composed of axons covered in myelin (Bastiani et al., 2012). Fibers within white matter connect different parts of the brain. These fibers, with greater myelin surrounding their axons, show more efficient communication between regions in the brain (Du et al., 2021). Magnetic resonance imaging (MRI) has a specific modality called diffusion-weighted imaging (DWI), which noninvasively measures the diffusion of water molecules within fibers in WMM and provides metrics of this diffusion, such as fractional anisotropy (FA) (Du et al., 2021). FA measures the degree of anisotropy of water molecules as they diffuse within thesewhite matter fibers and therefore provides a marker of WMM organization and orientation (Du et al., 2021). Across the lifespan, WMM develops rapidly over the first three years of life in concordance with the acquisition of cognitive skills and reaches a maximum in the second or third decade of life (Lebel and Deoni, 2018). In older adults, studies examining WMM changes find continued plasticity in WMM across the older adult lifespan and are related to cognitive functions, such as episodic memory (Merenstein et al., 2021).

A few studies have investigated WMM organization in human and animal parents across the transition to parenthood (Chan et al., 2015, Hoekzema et al., 2022, Voldsbekk et al., 2021). A recent DWI rodent study showed that pregnancy is associated with higher FA (Chan et al., 2015). Further, a study using diffusion imaging data from the UK Biobank found that older women who had one to three children showed “younger-looking” global and regional white matter compared to older adult women who were nulliparous (Voldsbekk et al., 2021). These studies indicate that childbearing, female parents experience increased organization in WMM from prenatal to the postpartum period. However, given that prior mentioned studies have exclusively examined female mammals, we are not certain how these results might replicate in fathers across the prenatal to the postpartum period. To date, only one study has examined WMM organization in fathers. This study investigated a sample of expectant and new fathers and found that higher WMM organization measured via FA, specifically in the uncinate fasciculus (UF), moderated the association between early life stress and handgrip strength (Alyousefi-van Dijk et al., 2021). Specifically, fathers and expectant fathers with higher FA in the UF had a less strong association between early life stress and stronger hand grip strength while hearing infant cry sounds. However, research has yet to examine the longitudinal reorganization of fathers’ brains during the transition to parenthood. The present study examines whether fathers show WMM organization changes across the prenatal to the postpartum period.

Though prior research on the adjustment to parenthood in parents has not extensively examined WMM organization, research examining gray matter (GM) volume points to plasticity in the parental brain across the transition to parenthood. Several studies have examined structural GM changes in the human maternal brain (Carmona et al., 2019, Hoekzema et al., 2017, Kim et al., 2018, Lisofsky et al., 2019). Although these studies all examine brain changes at different time points during the gestational period, they have found converging evidence for changes in GM volume (Martínez-García et al., 2021) in regions that overlap with the default mode network (e.g., medial prefrontal cortex and precuneus), the salience network (e.g., anterior cingulate cortex and insula), and subcortical regions (e.g., amygdala, thalamus, caudate, hippocampus, and nucleus accumbens) (Martínez-García et al., 2021). Only two longitudinal MRI studies have examined structural changes in the GM volume of fathers (Kim et al., 2014, Paternina-Die et al., 2020). Though the results of these two studies were somewhat distinct, both studies found GM volume reductions in the precuneus, a region of the brain involved in mentalizing (Kim et al., 2014, Paternina-Die et al., 2020). The present study seeks to provide complementary information to the existing GM literature by examining changes in the white matter of new parents.

Previous studies on parents' white matter changes across the transition to parenthood have yielded mixed results. Human research examining white matter across the transition to parenthood has typically examined changes in white matter (WM) volume in mothers. For instance, Zhang et al. (2019) detected greater increases in WM volume within the insula, postcentral gyrus, inferior parietal, and superior and middle temporal gyri during late postpartum in a sample of primiparous mothers (Martínez-García et al., 2021, Zhang et al., 2019). In contrast, other studies have failed to find changes in gyral WM thickness (Carmona et al., 2019) or WM volume (Hoekzema et al., 2017) when comparing mothers’ brain volume across the prenatal and postpartum periods. Inconsistent findings in prior studies examining WM volume may reflect the limitations of WM volume as a metric for detecting changes in WM. Prior research indicates the association between WM volume and FA are moderate-to-weakly correlated and that FA is a more sensitive value of WM changes (Fjell et al., 2008). Future examinations on white matter changes across the transition to parenthood in fathers can benefit from using DWI metrics, such as FA, that provide more nuanced information about white matter fiber changes. Specifically, in the present study, we examined fathers' WMM organization via FA.

Some evidence suggests that WMM organization may be linked with parental mental health. Prior evidence suggests that depression (Silver et al., 2018; Y. Wang et al., 2012) and stress (Fani et al., 2022, Meng et al., 2021) are associated with lower WMM organization as measured by FA. Regarding depression, Silver and colleagues (2018) examined WMM organization cross-sectionally in a sample of new mothers at 2–8 weeks postpartum. The study found women with postpartum depression, compared with healthy postpartum women, had significantly lower WMM in the left anterior limb of the internal capsule at 2–8 weeks postpartum (Silver et al., 2018). Further, women reporting more postpartum depression symptoms were more likely to have lower WMM within the corpus callosum. Regarding stress, Meng and colleagues (2021) examined longitudinal changes in WMM in a sample of people exposed to a large-scale earthquake (Meng et al., 2021). The study found that, compared to a demographically matched group of healthy controls, individuals exposed to the earthquake showed lower FA in parietal portions of the left superior longitudinal fasciculus and higher FA in the parietal portion of the left corticospinal tract. Further, individuals reporting reduced levels of anxiety and depression showed increased FA of the left uncinate fasciculus and left corticospinal tract. Given that fathers are also at risk of mental health problems (Kim and Swain, 2007, Saxbe et al., 2018) during the adjustment to parenthood, future studies can examine how fathers' WMM organization relates to mental health trajectories across the transition to parenthood. In the present study, we examined how changes in fathers' WMM organization via FA related to their depression and stress.

Using data from a study of new fathers transitioning to first-time parenthood, we examined changes in WMM organization from prenatal to postpartum at the whole brain and tract-specific level. In terms of specific tracts, we examined changes in the internal capsule, corpus callosum, longitudinal fasciculus, uncinate fasciculus, and corticospinal tract. We tested associations between changes in WMM organization at the whole brain and tract-specific level and mental health symptoms at six months postpartum. We had two hypotheses. First, we expected fathers would show reductions in global WMM organization from prenatal to postpartum at the whole brain and tract-specific level (i.e., internal capsule, corpus callosum, bilateral longitudinal fasciculus, bilateral uncinate fasciculus, and bilateral corticospinal tract). Second, we expected fathers who underwent more pronounced reductions in global WMM organization from the prenatal to postpartum period were likely to face increased difficulties in adjusting to parenthood, as evidenced by higher levels of depression, perceived stress, and parenting stress six months after the birth of their child. To ensure our results are rigorous, we corrected for multiple comparisons.

## 2 Materials and methods

### 2.1 Participants

**Fig. 1:**
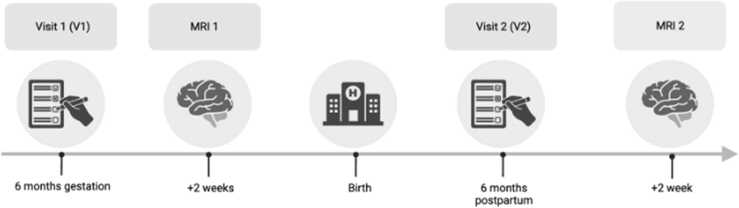
Timeline of Hormones and Attachment across the Transition to Childrearing (HATCH) study visits. Visit 1 (V1) took place at six months gestation. MRI1 took place two weeks after Visit 1. Visit 2 (V2) took place at six months postpartum. MRI 2 took place two weeks after Visit 2.

**Fig. 2:**
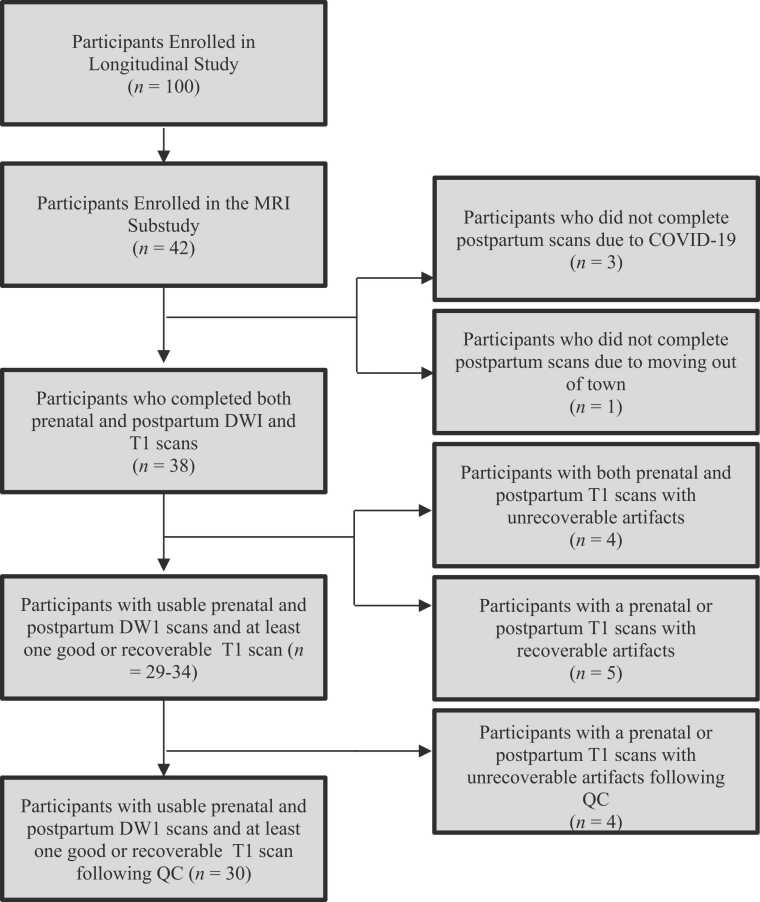
Flow chart describing the selection of participants for the current study.

Data from this study were drawn from a larger longitudinal study of the transition to parenthood (*n* = 100) that was approved by the Institutional Review Board at USC. The study also included an MRI substudy. For an overview of the longitudinal study, see Fig. 1. A total of 42 fathers enrolled in the MRI substudy and completed prenatal diffusion weighted imaging (DWI) and structural scans. Of the 42 fathers, four fathers did not complete their postpartum scan. Of these four, three of the fathers did not complete the postpartum scan because they were reluctant to return to the lab during the COVID-19 pandemic. The remaining father did not complete the postpartum scan because he and his family moved out of state. Thus, a total of 38 fathers completed both DWI and structural scans. Of the 38 fathers who completed both prenatal and postpartum scans, four fathers were excluded due to T1 scans with unrecoverable artifacts. Also, five fathers had at least one T1 scan with recoverable artifacts. The Stage I registered report projected a final sample between 29 and 34 fathers with both DWI scans and at least one T1 scan. For a visual flow chart describing the selection of participants for the current study, see Fig. 2.

Ultimately, at Stage II of the registered report, we excluded four participants due to unrecoverable T1 scan artifacts. We present descriptive statistics of the largest projected final sample (*n* = 34) and the final sample (*n* = 30) in Table 1. To examine whether the final sample was significantly different from the four individuals excluded due to T1 scan artifacts, we ran a series of independent sample t-tests between fathers’ age at the prenatal scan, the gestational age of the fetus at the prenatal scan, and infant age at the postpartum scan. An independent samples t-test revealed a statistically significant difference in the mean infant age at the postpartum scan between the four individuals excluded from the final registered report (*M* = 6.75 months) and the final sample of 30 (*M* = 9.11 months), suggesting that the fathers in the final sample had older infants at the postpartum MRI compared to the four fathers excluded, *t*(31.99) = −3.03, *p* =.005.

**Table 1:** Participant Demographics based on Substudy.

Variable		Total Sample	Final Sample
Sample size (#)		34	30
Father age at prenatal lab visit (years)		31.91 (4.16)	31.6 (3.65)
Fetal age at prenatal lab visit (months)		6.97 (1.09)	7.01 (1.14)
Infant age at postpartum lab visit (months)		8.84 (3.91)	9.11 (4.08)**
Ethnicity (%)	White	9 (26.47%)	7 (23.33%)
	Black/African American	3 (8.82%)	3 (10.00%)
	Hispanic or Latino/a	11 (32.36%)	10 (33.33%)
	Asian or Pacific Islander	9 (26.47%)	8 (26.67%)
	Other	2 (5.88%)	2 (6.67%)
Education (%)	Some college	6 (17.65%)	5 (16.67%)
	Associate's degree	1 (2.94%)	1 (3.33%)
	Bachelor's Degree	12 (35.29%)	11 (36.67%)
	Master's degree	10 (29.41%)	9 (30.00%)
	Doctoral degree	5 (14.71%)	4 (13.33%)

*Note*. An independent samples t-test revealed a statistically significant difference in the mean infant age at the postpartum visit between the four individuals excluded from the registered report and the final sample of 30 included in the registered report such that those included in the final sample had infants who were significantly older at the postpartum MRI. * <.05; ** <.01; ***<.001

At one year postpartum, participants were asked to retrospectively report their use of leave time.

The largest percentage of participants (46.67%, *n* = 14) in the final sample took between 1 and 3 months of leave. Of the remaining participants, 13.33% (*n* = 4) took less than one month of leave, 6.67% (*n* = 2) took 4–6 months of leave, 10.0% (*n* = 3) took less than 6 months of leave, and 6.67% (*n* = 2) took more than 9 months of leave. Finally, 6.66% (*n* = 5) of participants did not provide any data on leave time. To examine whether cohort status (i.e., pre-COVID, Post-COVID) was related to participants’ use of leave time (i.e., 5 levels for months of leave), we conducted a chi-square test of independence. Of the participants reporting their use of leave time results did not indicate a significant association, *χ²*(4, *n* = 25) = 3.50, *p* =.48.

### 2.2 Procedure

**Fig. 3:**
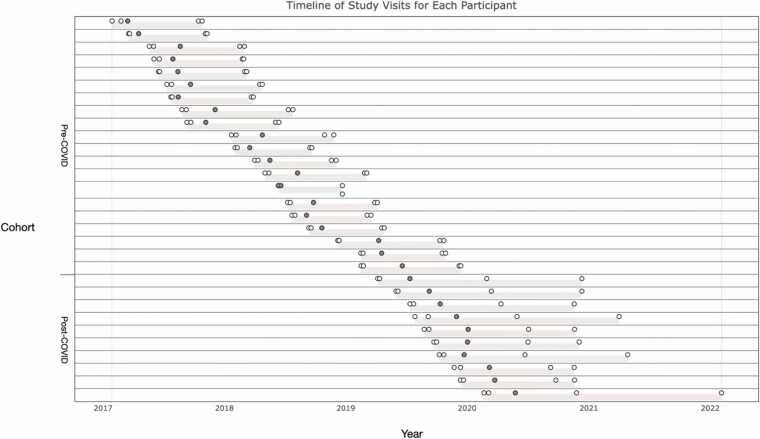
Timeline of study visits for each participant. The x axis depicts the year during which the study visits took place and the y axis represents whether the participants did their postpartum scan before or during COVID-19 pandemic lockdowns. The first twenty participants were in the Pre-pandemic cohort and the last ten participants were in the Pandemic cohort. Each horizontal line represents one participant’s timeline of visits. The individual circles within each line represent the individual events of interest for the study: prenatal lab visit, prenatal MRI visit, birth, postpartum lab visit, and postpartum MRI visit. All study visits are depicted as white circles and gray circles represent the date of birth of the infant.

Fig. 1 outlines the timeline of study visits. During the mid-to-late pregnancy visit (i.e., V1; *M* = 6.64, *SD* = 1.17) and six months postpartum (i.e., V2; *M* = 6.41, *SD* = 0.50) lab visits, each couple (i.e., father and mother) completed questionnaires including assessments of depressive symptoms and perceived stress. Fathers were also sent a link to complete questionnaires online at 12 months following their infant's birth. Questionnaires measuring parenting stress and postpartum depression were added at the postpartum visit. The prenatal (i.e., MRI1; *M* = 7.01, *SD* = 1.14) and postpartum MRI substudy (i.e., MRI2; *M* = 9.11, *SD* = 4.08) visits were scheduled two weeks after V1 and V2, respectively. During MRI1 and MRI2, fathers completed a diffusion-weighted imaging (DWI) scan and a structural scan. Of note, given the COVID-19 pandemic, ten postpartum MRI scans were delayed by lockdown restrictions (see Fig. 3). As such, the timing of the self-report questionnaires and the postpartum MRI was delayed for the last ten fathers. More specifically, the time between V2 and MRI2 was shorter for the Pre-pandemic cohort compared to the Pandemic cohort. Given this discrepancy, we utilized self report questionnaires from a 12 months postpartum follow up questionnaire for seven of the ten fathers in the Post-COVID cohort whose infants were roughly 1 year in age (*M* = 12.13 months).

## 3 Measures

We present mean, standard deviation, and range of included measures in Table 2.

**Table 2:** Study measures.

Variable	*M*	*SD*	*Range*	*p*
Prenatal Beck Depression Inventory	7	7.28	(0−37)	.98^^^
Postpartum Beck Depression Inventory	6.97	5.14	(1−23)	
Prenatal Perceived Stress Scale	21.1	6.77	(9−37)	.29^^^
Postpartum Perceived Stress Scale	19.37	5.59	(4−29)	
Parenting Stress Index	79.07	18.86	(50.67–113.33)	
Edinburgh Postnatal Depression Scale	3.7	2.94	(0−9)	

*Note*. *M* = mean; *SD* = standard deviation; *Range* = range; *p* = p-value. All postpartum scores reflect the postpartum questionnaire closest to the postpartum MRI scan. ^ = Two paired samples t-tests revealed no statistically significant difference in the mean prenatal BDI and postpartum BDI scores, and no difference between mean prenatal PSS and postpartum PSS scores.

### 3.1 Depression

The Beck Depression Inventory- II (BDI-2), administered at both the prenatal and postpartum visits, is a self-report multiple-choice measure of depressive symptoms in the past two weeks (Beck et al., 1996), with 21 items rated on a 4-point Likert scale (0 = No severity; 3 = High severity). The BDI-2 assesses depressive symptoms (e.g., hopelessness, irritability) and physical symptoms (e.g., fatigue, weight loss). BDI-2 yields a total score ranging from 0 to 63, and higher scores indicating greater depressive symptoms. Prior research concludes that the BDI-2 has high reliability and validity (Y.-P. Wang and Gorenstein, 2013). In the present sample, Cronbach’s alpha for the prenatal BDI was α =.95, 95% CI [0.94, 0.96], six months postpartum BDI was α =.95, 95% CI [0.94, 0.96], and 12 months postpartum BDI was α =.77, 95% CI [.68,.83].

### 3.2 Perceived Stress

The Perceived Stress Scale-14 (PSS-14) (Cohen et al., 1983), also administered at both prenatal and postpartum, has 14 items rated on a 5-point Likert scale (0=Never; 4=Very often). The PSS-14 detects whether participants perceived situations in the past month as stressful, with total scores ranging from 0 to 56 and higher scores indicating greater perceived stress. Prior evidence suggests that the PSS-14 has high reliability and validity (Cohen, 1988)). In the present sample, Cronbach’s alpha for 6 months prenatal PSS was α =.84, 95% CI [0.77, 0.88], 6 months postpartum PSS was α =.75, 95% CI [0.72, 0.76], and 12 months postpartum as α =.75, 95% CI [0.73, 0.76].

### 3.3 Postpartum Depression

The Edinburgh Postnatal Depression Scale (EPDS) (Cox et al., 1987), administered at the postpartum visit, has ten items rated on a 4-point Likert scale (0=Never; 3 = Yes, most of the time). The EPDS detects how often participants perceived situations in the past 7 days, with total scores ranging from 0 to 30 and higher scores indicating greater risk for postpartum depression. At the university IRB request, a question on suicidality was dropped. Previous research indicates that the EPDS has high reliability and validity in parent samples (Matthey et al., 2001, Shrestha et al., 2016). In the present sample, Cronbach’s alpha for the six months EDPS was α =.82, 95% CI [.73,.87] and 12 months EDPS was α =.79, 95% CI [.73,.84]

### 3.4 Parenting Stress

The Parenting Stress Index-Fourth Edition-Short Form (PSI-4-SF), administered at the postpartum visit, was designed for parents of children ranging from 1 month to 12 years. The PSI-4-SF has 36 items and uses a 5-point Likert scale (1 = Strongly agree; 5 = Strongly disagree). The PSI-4-SF identifies issues that may lead to problems in the child’s or parent’s behavior, including parental distress (i.e., the extent of stress felt in the role as a parent), parent-child dysfunctional interaction (i.e., the extent to which the parent believes that his/her child does not meet their expectations and their interactions are not satisfying), and difficult child (i.e., how easy or difficult the parent perceives the child).The PSI-4-SF yields a total score ranging from 0 to 100, with higher scores indicating a higher degree of stress in the parent-child system. Prior studies suggest that the PSI-4-SF has high reliability and validity (Haskett et al., 2006, Subica et al., 2014). In the present sample, Cronbach’s alpha for the six months PSI-4 was α =.93, 95% CI [.90,.95] and 12 months PIS-4 was α =.95, 95% CI [.93,.96].

### 3.5 Image Acquisition

We collected data using a 3.0 Tesla Siemens MAGNETOM Prisma^fit^ scanner with a 20-channel head coil. At both prenatal and postpartum scans, we collected a high-resolution MPRAGE and whole brain DTI. We acquired the high-resolution MPRAGE scans with the following parameters: repetition time [TR] = 2530 ms; echo time [TE] = 3.13 ms; flip angle = 10°; voxel dimensions = 1 ×1×1 mm. We acquired the whole brain DTI data with the following parameters: 70 contiguous slices were collected in axial orientation; voxel dimension = 2 mm×2 mm x 2 mm; repetition time; TR = 8100 ms; echo time, TE = 69.0 ms; fat saturation, on; frequency direction, anterior/posterior; field of view = 256 mm×256 mm; in-plane resolution 2 mm. A baseline image *b* value of 0 s/mm^2^ and 64 different diffusion orientations were acquired with a *b* value of 1000 s/mm^2^. Total acquisition time for the DTI protocol was 9 minutes and 45 seconds. Throughout the scan, we instructed participants to remain awake and were presented with a standard fixation cross.

### 3.6 Statistical analysis

#### 3.6.1 Power analysis

Power analysis conducted in G*Power (Faul et al., 2007, 2009) for mean differences (Hypothesis 1) showed that our final sample of 30 participants with prenatal and postpartum scans had 85% power to detect a medium-sized effect (Cohen's *d* = 0.5) and 99% power to detect a large-sized effect (Cohen's *d* = 0.8). Power analysis for regression analyses for our final sample of 30 participants with mental health variables (Hypothesis 2) showed that our power analysis showed 91% power to detect a medium effect (Cohen's *d* = 0.5) with four predictors) and 99% power to detect a large-sized effect (Cohen's *d* = 0.95). Using Cohen’s *f*^*2*^, a common measure of effect size using R^2^, our whole-brain effect size for prenatal to postpartum increase was.50 and prenatal to postpartum decrease was.60. These effect sizes are consistent with our projections and suggest that we had adequate power to conduct the study. We have also reported the standardized regression coefficients to show effect sizes of all covariates included in the models (see Supplemental Table 2).

#### 3.6.2 DWI quality checking

Diffusion-weighted images were processed with DTIprep to automatically detect common artifacts, correct for motion, and eddy current deformations, and exclude bad gradients (Liu et al., 2010, Oguz et al., 2012). Following quality checking, we were able to maintain 30 participants with sufficient gradients (>18 gradients).

#### 3.6.3 DWI preprocessing

Additional DWI preprocessing was performed using DSI Studio (http://dsi-studio.labsolver.org/; 2021, 2022) to prepare for connectometry analyses. The b-table was checked by an automatic quality control routine to ensure its accuracy (Schilling et al., 2019). For quality check, fiber orientations were compared to the population-averaged template (Yeh et al., 2018). The b-table was flipped by.012fy. The diffusion data were reconstructed in the MNI space using q-space diffeomorphic reconstruction (Yeh et al., Neuroimage, 58(1):91–9, 2011) to obtain the spin distribution function (Yeh et al., IEEE TMI,;29(9):1626–35, 2010).

A diffusion sampling length ratio of 1.25 was used. The output resolution in diffeomorphic reconstruction was 2 mm isotorpic. The tensor metrics, namely FA, were calculated using DWI with b-value lower than 1750 s/mm².

Goodness-of-fit with an R^2^ statistic between each participant’s quantitative anisotropy (QA) map and MNI QA was examined; a cutoff of R^2^ > 0.5 was used for quality assurance based on DSI developer recommendations and previous studies (Hodgdon et al., 2022). All participants QA maps had R^2^ statistics above 0.71.

#### 3.6.4 Hypothesis 1

Correlational tractography (Yeh Neuroimage 2021) was performed using DSI Studio (http://dsi-studio.labsolver.org/; 11.16.2023) to study longitudinal changes in FA. The anisotropy threshold was randomly selected. A total of 1000000 seeds were placed at various regions of the brain. Topology-informed pruning (Yeh et al., 2019) was applied to the tractography with 4 iteration(s) to remove false connections (Hodgdon et al., 2022; Sihvoenen et al., 2021; Zheng et al., 2022) and a T-score threshold of 2 (Hodgdon et al., 2022; Sihvonen et al., 2021). An FDR connection of 0.05 was used to select tracts. We performed the analysis first in the whole brain and next in the specific tracts (i.e., internal capsule, corpus callosum, longitudinal fasciculus, uncinate fasciculus, and corticospinal tract) where we hypothesized that changes would occur. Brain areas were segmented based on HCP842 Tractography Atlas. Permutation-based multiple comparison corrections were applied to determine statistical significance (Nichols and Holmes, 2002). We corrected for multiple comparisons using permutation testing with a total of 8000 randomized permutations (Yeh et al., 2017). For whole brain analyses, we repeated the tract-specific analyses on the whole brain. We corrected for multiple comparisons using permutation testing with a total of 2000 randomized permutations.

#### 3.6.5 Hypothesis 2

Correlational tractography (Yeh Neuroimage 2021) was performed using DSI Studio (http://dsi-studio.labsolver.org/; 11.16.2023) to calculate the association between FA difference from the prenatal scan and postpartum scan with the adjustment to parenthood variables (depression, stress, and parenting stress) adjusting for fathers' age, the gestational age of the fetus at the prenatal time point, and the age of the infant at the postpartum time point.

We conducted correlational tractography within specific tracts where we hypothesized changes would occur. In addition, for a more data driven approach, we ran the analysis at the whole brain level to identify tracts not previously hypothesized. For tract-specific analyses of previously hypothesized areas (i.e., internal capsule, corpus callosum, longitudinal fasciculus, uncinate fasciculus, and corticospinal tract), we selected our ROI in the MNI space by selecting study regions from the HCP842 Tractography atlas for each ROI. We used multiple regression to identify GFA-based local connectomes within each specific tract that were significantly associations with the adjustment to parenthood variables (i.e., depression, stress, parenting stress, postpartum depression adjusting for fathers' age, the gestational age of the fetus at the prenatal time point, and the age of the infant at the postpartum time point). As in Hypothesis 1, topology-informed pruning (Yeh et al., 2019) was applied to the tractography with 4 iteration(s) to remove false connections (Hodgdon et al., 2022; Sihvoenen et al., 2021; Zheng et al., 2022) and a T-score threshold of 2 (Hodgdon et al., 2022; Sihvonen et al., 2021). We corrected for multiple comparisons using permutation testing with a total of 8000 randomized permutations (Yeh et al., 2017). For whole brain analyses, we repeated the tract-specific analyses on the whole brain. We corrected for multiple comparisons using permutation testing with a total of 2000 randomized permutations.

## 4 Results

### 4.1 Examining normality in study covariates

Kolmogorov-Smirnov tests (Olea and Pawlowsky-Glahn, 2009) were used to test for normality on the main study covariates. For the one-sample Kolmogorov-Smirnov tests, we found that the distribution of residuals was significantly different from a normal distribution for fathers’ age at the prenatal lab visit (*D* = 1, *p* <.001), gestational age of the fetus at the prenatal MRI visit (*D* = 1, *p* <.001), and infant age at the postpartum MRI visit (*D* = 1, *p* <.001). In order to maintain normality assumptions, we z-transformed each study variable.

### 4.2 Examining differences in data collection timing due to COVID-19 lockdowns

**Fig. 4:**
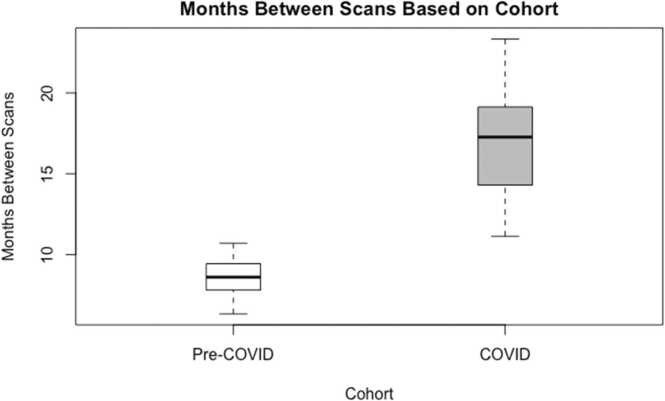
Bar graphs depicting the months between prenatal and postpartum scans as a function of cohort membership.

As mentioned in the Methods, the timing of the second scan was affected by COVID-19-related lockdowns for the last 10 fathers in the sample. A two sample t-test was performed to compare the number of days between the prenatal and postpartum scans based on whether the participants completed both scans prior to the COVID pandemic (i.e., Pre-pandemic cohort) or completed the postpartum scan after the COVID pandemic began (i.e., Pandemic cohort). There was a significant difference in the number of days between prenatal and postpartum scans for the Pre-pandemic cohort (*M* = 258.65) and Pandemic cohort (*M* = 501.40); *t*(9.74) = −6.40, *p* <.001 (Fig. 4). Given the large distribution in follow-up scan timing, we controlled for cohort status in all analyses (i.e., Pre-pandemic vs Pandemic).

### 4.3 Effect of length threshold on whole brain results

We conducted whole brain analyses using varied tract lengths, specifically 5, 10, 15, 20, 25, and 30 voxels. After examination of preliminary results (Supplemental Fig. 1), we continued with the tract length of 15 voxels. This length provided precision and robustness in assessing changes concurrently with other parameters.

### 4.4 Primary analyses

#### 4.4.1 Changes in FA from prenatal to postpartum

**Fig. 5:**
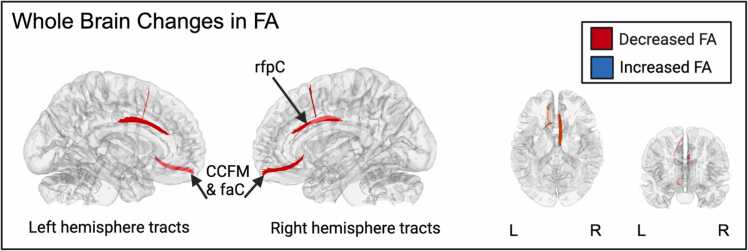
Connectometry results of whole brain longitudinal changes in FA. Red represents tracts showing decreased FA. Blue represents tracts showing increased FA. Five tracts are labeled. The cerebellum was excluded from the analysis and the seeding region was placed at the whole brain. T-score = 2, FDR < 0.05. FA = Fractional anisotropy, L = Left, R = Right, rfpC = Right frontal parietal cingulum, CCFM = Corpus callosum forceps minor, Frontal anterior commissure = faC.

**Results from Whole Brain.** We ran a whole-brain DMRI connectometry model to identify areas with changes in white matter FA from prenatal to postpartum, adjusting for participant’s age at the prenatal MRI visit, gestational age of the fetus at the prenatal MRI visit, age of the infant at the postpartum MRI visit, and cohort (Pre-pandemic vs Pandemic). The connectometry analysis identified FA decreases in several tract bundles (FDR =.002, Fig. 5), including right frontal parietal cingulum, corpus callosum forceps minor, and the frontal anterior commissure. The full list of tract bundles with FA decreases from prenatal to postpartum are listed in the supplemental materials along with their respective FDRs (Supplemental Table 3).

**Fig. 6:**
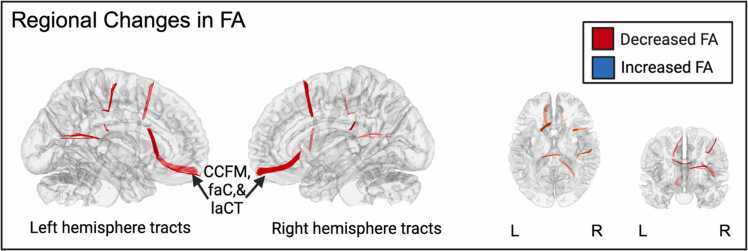
Connectometry results of longitudinal changes in white matter from prenatal to postpartum in tracts that travel through the Corpus Callosum. Red represents tracts showing decreased FA. Three tracts are labeled. The cerebellum was excluded from the analysis and the seeding region was placed at the whole brain. T-score = 2, FDR < 0.05. An ROI was placed at the corpus callosum. Decreases in white matter tracts from prenatal to postpartum. FA = Fractional anisotropy, L = Left, R = Right, CCFM = Corpus callosum forceps minor, faC = Frontal anterior commissure, laCT = Left anterior cortico-striatal tract.

**ROI Results.** We ran individual multiple regression models on five ROIs (corpus callosum, corticospinal tract, internal capsule, longitudinal fasciculus, uncinate fasciculus) in DMRI connectometry to identify areas with changes in white matter FA from prenatal to postpartum, adjusting for participant’s age at the prenatal MRI visit, gestational age of the fetus at the prenatal MRI visit, age of the infant at the postpartum MRI visit, and cohort (Pre-pandemic vs Pandemic). The ROI connectometry analysis identified reduced connectivity in several white matter tracts passing through the corpus callosum (FDR =.001, Fig. 6), including corpus callosum forceps minor, frontal anterior commisure), and left anterior cortico-striatal tract. List of full regions with reduced connectivity are listed in the supplemental materials (Supplemental Table 4).

The ROI connectivity analyses for the corticospinal tract, internal capsule, longitudinal fasciculus and uncinate fasciculus identified no changes in white matter FA from prenatal to postpartum.

#### 4.4.2 Association between postpartum mental health and change in FA from prenatal to postpartum

##### 4.4.2.1 Whole brain results

We measured postpartum depression using two different measures, the BDI-II and the EPDS, and found slightly different patterns of results for each.

**Fig. 7:**
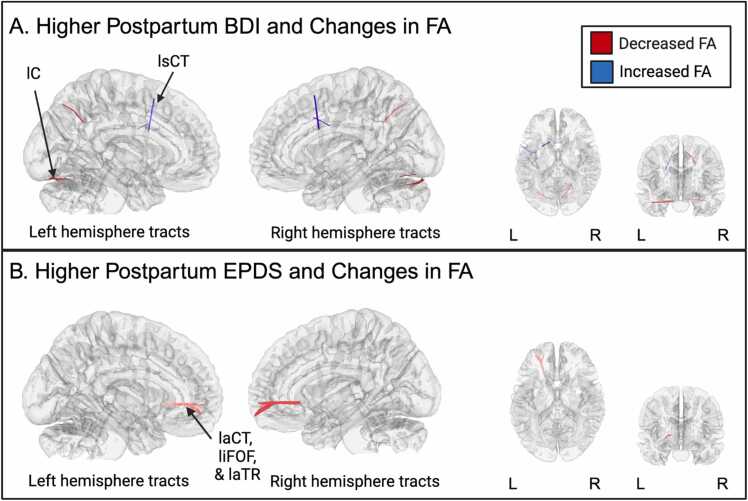
Connectometry results of association between higher BDI and longitudinal changes in white matter from prenatal to postpartum. Red represents tracts showing decreased FA. Blue represents tracts showing increased FA. The cerebellum was excluded from the analysis and the seeding region was placed at the whole brain. T-score = 2., FDR < 0.05. An ROI was placed at the corpus callosum. (A) Association between higher BDI and longitudinal FA changes. Two tract bundles labeled. (B) Association between higher EPDS and longitudinal changes. Three tracts labeled. FA = Fractional anisotropy, L = Left, R = Right, lC = Left cerebellum, lsCT = Left superior cortico-striatal tract, laCT = Left anterior cortico-striatal tract, liFOF = left inferior fronto occipital fasciculus, laTR = Left anterior thalamic radiation.

Connectometry analyses revealed several tracts in which higher depressive symptoms at postpartum as measured by the BDI-II were associated with FA increases in several fiber bundles, including left superior cortico-striatal tract (FDR =.043; Fig. 7A). Connectometry analyses revealed tract bundles in which higher postpartum depression symptoms at postpartum as measured by the BDI-II were associated with FA decreases in left cerebellum (FDR =.026; Fig. 6A). The full list of tract bundles and their respective FDRs are reported in Supplemental Table 5.

Connectometry analyses revealed several tracts in which higher symptoms of postpartum depression as measured by the EPDS were associated with decreases in FA values in several regions (FDR =.004; Fig. 7B), including left anterior cortico-striatal tract, left inferior fronto-occipital fasciculus, and left anterior thalamic radiation. FDR for each tract bundle is reported in Supplemental Table 6.

The whole brain analyses yielded no significant changes in FA from prenatal to postpartum associated with parenting stress or postpartum perceived stress.

##### 4.4.2.2 ROI results

Given that we found no significant longitudinal changes in white matter FA tracts that pass through the corticospinal tract, internal capsule, longitudinal fasciculus, and uncinate fasciculus, we only conducted ROI analyses in the corpus callosum. Specifically, we conducted DMRI connectometry to identify tracts with changes in white matter FA that passed through the corpus callosum and were linked with postpartum mental health (depression, stress, or parenting stress), adjusting for participant’s age at the prenatal MRI visit, gestational age of the fetus at the prenatal MRI visit, age of the infant at the postpartum MRI visit, and cohort.

##### 4.4.2.3 Corpus callosum ROI results for postpartum depression

**Fig. 8.**
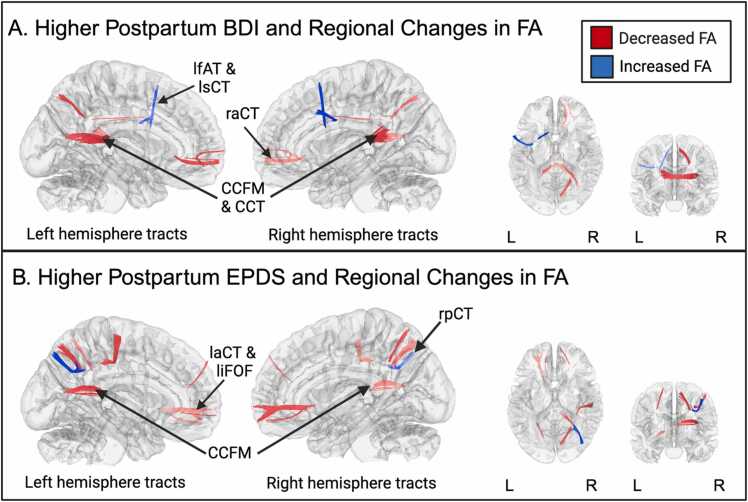
Connectometry results of longitudinal changes in white matter from prenatal to postpartum in tracts that travel through the Corpus Callosum associated with postpartum depression measured by BDI and EPDS. Red represents tracts showing decreased FA. Blue represents tracts showing increased FA.Three tracts are labeled. The cerebellum was excluded from the analysis and the seeding region was placed at the whole brain. T-score = 2, FDR < 0.05. FA = Fractional anisotropy, L = Left, R = Right. (A) Association between higher BDI and longitudinal FA changes. (B) Association between higher EPDS and longitudinal changes. L = Left, R = Right, lfAT= Left frontal aslant tract, lsCT= Left superior cortico-striatal tract, raCT= Right anterior cortico-striatal tract, CCFM=Corpus callosum forceps major, CCT=Corpus callosum tapetum, rpCT = Right posterior cortico-striatal tract, laCT= Left anterior cortico-striatal tract, liFOF= Left inferior fronto occipital fasciculus.

Connectometry analyses revealed several tracts in which higher depressive symptoms at postpartum as measured by the BDI-II were associated with FA increases in several regions that pass through the corpus callosum (FDR =.002; Fig. 8A), including left frontal aslant tract and left superior cortico-striatal tract. FDR for each tract bundle is reported in Supplemental Table 7. Connectometry analyses revealed several tracts in which higher postpartum depression symptoms as measured by the BDI-II were associated with FA decreases in several regions that pass through the corpus callosum (FDR =.001; Fig. 8A), including corpus callosum forceps major, corpus callosum tapetum, and right anterior cortico-striatal tract. FDR for each tract bundle is reported in Supplemental Table 7.

Connectometry analyses revealed several tracts in which higher postpartum depression symptoms as measured by the EPDS were associated with FA increases in several regions that pass through the corpus callosum (FDR =.014;Fig. 8B), including right posterior cortico-striatal tract. FDR for each tract bundle is reported in Supplemental Table 8. Connectometry analyses revealed several tracts in which higher postpartum depression as measured by the EPDS were associated with FA decreases in several regions (FDR =.000; Fig. 7B), including corpus callosum forceps major, left inferior fronto occipital fasciculus, and left anterior cortico-striatal tract. FDR for each tract bundle is reported in Supplemental Table 8.

##### 4.4.2.4 Corpus callosum ROI results for perceived stress

The corpus callosum ROI analyses yielded no significant changes in FA from prenatal to postpartum associated with perceived stress.

##### 4.4.2.5 Corpus callosum ROI results for parenting stress

The corpus callosum ROI analyses yielded no significant changes in FA from prenatal to postpartum associated with parenting stress.

### 4.5 Follow-up analyses

#### 4.5.1 Examining covariates

In order to determine whether the covariates impacted our primary findings at the whole brain level from prenatal to postpartum, we reanalyzed the data with and without the stated covariates: the effect of paternal prenatal age (years), gestational age at the prenatal scan (days), and time from delivery at the postpartum scan (days), and cohort (Pre-pandemic vs Pandemic). Results were substantially unchanged.

#### 4.5.2 Examining cohorts

**Fig. 9:**
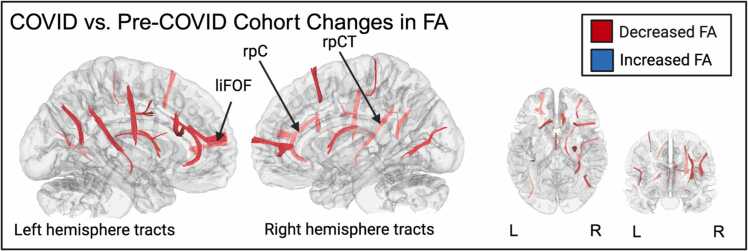
Differences between COVID and Pre-COVID cohorts in longitudinal FA changes (*n*= 30). Red represents decreased FA. The cerebellum was excluded from the analysis and the seeding region was placed at the whole brain. T-score = 2 FDR < 0.05. FA = Fractional anisotropy, L = Left, R = Right, liFOF = Left inferior fronto occipital fasciculus, rpC = Right parolfactory cingulum, rpCT = Right posterior cortico-striatal tract.

To further probe the effect of cohort, we reanalyzed the whole-brain model with cohort as the primary variable of interest (*n* = 30). The COVID cohort showed greater FA decreases compared to the Pre-COVID cohort in several regions (FDR =.000, Fig. 9), including right parolfactory cingulum, right posterior cortico-striatal tract, and left inferior fronto occipital fasciculus. The full list of regions with FA decreases and their respective FDRs are listed in the supplemental materials (Supplemental Table 9).

## 5 Discussion

This study represents the first examination of changes in white matter microstructure (WMM) organization across the transition to first-time fatherhood. The study also examines whether changes in WMM organization were related to men’s postpartum mental health. We found that first-time fathers exhibited reduced FA at the whole-brain level, especially in the cingulum, a key tract connecting the limbic lobe with the neocortex (Maldonado et al., 2020) and associated with emotional regulation (Hung et al., 2020) as well as episodic memory (Bubb et al., 2018). We also found that first-time fathers displayed reduced FA at the regional level of the corpus callosum, especially in the forceps minor, which is implicated in cognitive functioning and attention (Mamiya et al., 2018). Changes in white matter FA were significantly correlated with postpartum depression but not perceived or parenting stress measures. Overall, our findings suggest that the transition to fatherhood is linked with changes in WMM organization and postpartum depressive symptoms.

At the whole brain level, we found reductions in WMM connectivity across multiple brain regions (e.g., right frontal parietal cingulum, corpus callosum forceps minor) across men’s transition to first-time fatherhood. As previously noted, the cingulum and corpus callosum forceps minor may support emotion regulation and executive functioning, respectively. A reduction in FA can be associated with many potential microstructural changes, including a reduced axonal alignment, axon density, and myelin density (Pujar et al., 2017). Some researchers have posited that gray matter volume decreases seen during the transition to motherhood may be related to cellular processes, namely synaptic pruning and gaining in myelinated white matter, supporting a theory of synaptic pruning paired with enhanced cellular communication (Carmona et al., 2019). Based on prior evidence of reductions in gray matter volumes in fathers, researchers have hypothesized that first time fathers would show a reduction in brain cells or increased WM myelination (Martínez-García et al., 2022). Our findings provide the first direct evidence that fathers show a reduction in FA, which we interpret as a general reorganization of the brain during the prenatal to postpartum transition. Similar to the whole brain results, our regional level results yielded FA reductions primarily in the corpus callosum forceps minor suggesting regional reorganization in an area that supports cognitive functioning and attention (Mamiya et al., 2018). These regional findings further suggest that the transition to fatherhood is accompanied by reorganization in key fibers that support cognitive processing.

We found positive and negative associations between multiple measures of postpartum depression and changes in patterns of FA. We operationalized postpartum depression using two widely used self-report questionnaires and found slightly different patterns of results for each. Higher postpartum depression symptoms as measured on the Beck Depression Inventory II (BDI-II) were associated with FA *increases* in regions supporting directed behavior (e.g., left superior cortico-striatal tract), as well as FA *decreases* in regions supporting coordinated movement and balance (e.g., left cerebellum). In contrast, higher postpartum depression symptoms measured via the Edinburgh Postnatal Depression Scale (EPDS) were associated with FA *decreases* in the left anterior cortico-striatal tract as well as areas supporting speech and language function (e.g., right fronto occipital fasciculus), and decision and emotion regulation (e.g., left anterior thalamic radiation). These findings replicate previous research that greater depression symptoms are associated with lower FA in the anterior thalamic radiation, a white matter bundle that may influence emotion regulation (Lai and Wu, 2014). Additionally, these findings are somewhat similar to previous connectometry studies associating microstructural organization in subcortical reward circuits (e.g., cortico-striatal tracts) and higher depression symptoms (Blood et al., 2010). However, we found slightly different FA patterns in distinct regions of the cortico-striatal tract depending on the measure of depression. Given that FA can increase and decrease as a function of myelin changes and structural coherence (Pujar et al., 2017), we interpret these mixed findings to reflect general structural reorganization of the cortico-striatal tract. These findings could reflect the distinct aspects of depression measured by each instrument (i.e., BDI-II and EPDS). Although the BDI-II has been validated to measure perinatal depression, the instrument includes items that examine changes in sleeping patterns and appetite, which may be common during early parenthood independent of depression (Brodey et al., 2016). In contrast, the EPDS was designed to examine depression symptoms within the context of the postpartum period. Thus, BDI-II may be assessing a more general presentation of depression while EPDS may be more sensitive to depression within the context of new parenthood. Overall, our findings fit with the existing cross-sectional literature in mothers linking greater postpartum depression (as measured by the EPDS) with lower WMM organization as measured by FA (Silver et al., 2018). Our findings suggest that fathers who report symptoms of depression in the postpartum period may show different patterns of longitudinal WMM reorganization across the transition to fatherhood, warranting greater investigation of the causal mechanisms underlying this association.

Changes in fathers’ WMM could be related to larger neurobehavioral changes during the transition to fatherhood. More specifically, these changes could be a result of neuroendocrine fluctuations, such as reductions in testosterone and estradiol across pregnancy and increases in oxytocin across the postpartum period (Bakermans-Kranenburg et al., 2019). Future research can explore the hormonal underpinnings of potential changes to WMM. Some research in marmoset males, whose fathers engage in extensive caregiving, suggests that increased parenting experience is associated with increased density of dendritic spines, facilitated by increases in vasopressin receptors (Kim et al., 2014, Kozorovitskiy et al., 2006). Furthermore, Kim et al. (2014) found that fathers’ greater paternal intrusiveness, operationalized via father’s physical contact with the infant during an in-lab father-infant play interaction, was associated with greater volume decreases across the postpartum period in the orbitofrontal cortex (OFC), a brain region that supports the learning of emotional value, emotional relevance, and uncertainty (Kim et al., 2014). In the current study, although we examined parenting stress, we were not able to test in-vivo parenting interactions or whether fathers’ everyday time devoted to caregiving was associated with WMM. Examining paternal interactions and investment more comprehensively, e.g. through behavioral play interactions and ecological momentary assessments, represents a future direction in parenting brain research.

The study has some additional limitations. First, while comparatively larger than other parenting brain studies that have been published, our sample size is still relatively small, constraining our ability to detect more nuanced changes in WMM. Although our sample was relatively diverse in terms of race/ethnicity and socioeconomic status, it was composed of fathers in heterosexual, cohabitating relationships who were able and willing to participate in a longitudinal study. Thus, our findings may not generalize to different family structures (e.g., divorced or non-residential fathers). Additionally, we assessed depression within a community sample of fathers rather than a clinical population, and operationalized mental health via self-report measures, which can be subject to social desirability biases. Future studies may benefit from focusing on high-risk populations and using other approaches, such as clinical interviews, to assess mental health. Finally, our longitudinal study design is exploratory in nature rather than explanatory, thereby limiting our ability to examine causality. Like the current study, randomization is rarely possible within studies of human pregnancy. However, future studies may benefit from utilizing a non-probabilistic sampling design that leverages an age matched control group of non-fathers.

Despite these limitations, this study has several notable strengths. To the best of our knowledge, this is the first study to examine changes in WMM organization from prenatal to postpartum among first-time fathers, thereby contributing to our understanding of the neurobiological changes associated with the transition to fatherhood. The use of FA as a measure of WMM organization is another strength of this study. By measuring FA, we build on prior studies on the transition to parenthood measuring WM thickness (Carmona et al., 2019) and WM volume (Hoekzema et al., 2017, Zhang et al., 2019), as well as the one longitudinal study of FA in first-time mothers (Hoekzema et al., 2022). FA is a more sensitive indicator of white matter changes and can provide more nuanced information about white matter changes (Fjell et al., 2008).

In conclusion, we found evidence for longitudinal changes in white matter microstructure organization in first-time fathers across the prenatal to postpartum periods. Our findings provide evidence that the transition to fatherhood is associated with neurobiological changes. Although positive father involvement is associated with better childhood outcomes, fathers are understudied in child development research. This research further emphasizes the value of translational neuroscience work that includes parents and caregivers beyond biological mothers.

Future directions for research include testing whether these white matter microstructure organization changes are temporary or enduring in ways that might have implications for men’s lifelong brain health. In addition, as hypothesized by key researchers in the parental brain field, the transition to fatherhood may reflect a neural specialization (Paternina-Die et al., 2020). As such, forthcoming research can assess whether white matter microstructure organization changes in fathers relate to men’s ability to parent effectively in the first years after the birth of a child. Given that both mothers and fathers experience notable shifts in sleep quality during the peripartum period, future studies should examine how sleep moderates white matter microstructure organization. Finally, future research can consider how additional contextual family stressors, such as access to family social support or paid family leave, shape fathers’ white matter microstructure organization.

## CRediT authorship contribution statement

**Fang-Cheng Yeh:** Writing – review & editing, Supervision, Software. **Pia E. Sellery:** Writing – review & editing, Project administration. **Van Truong:** Writing – review & editing, Visualization, Project administration, Data curation. **Yael H. Waizman:** Writing – review & editing, Data curation. **Sofia Isabela Cárdenas:** Investigation, Funding acquisition, Formal analysis, Data curation, Conceptualization. **Vidya Rajagopalan:** Writing – review & editing, Writing – original draft, Supervision, Methodology, Investigation, Formal analysis, Data curation. **Sarah A. Stoycos:** Writing – review & editing, Project administration. **Darby E. Saxbe:** Writing – review & editing, Writing – original draft, Supervision, Methodology, Investigation, Funding acquisition, Conceptualization.

## Declaration of Competing Interest

The authors declare that they have no known competing financial interests or personal relationships that could have appeared to influence the work reported in this paper.

## Data Availability

Data will be made available on request.
