# Supplementary material for: White matter microstructure organization across the transition to fatherhood
Source: Dev Cogn Neurosci. 2024 Apr 12;67:101374. doi: 10.1016/j.dcn.2024.101374 (PMC11021911; doi:10.1016/j.dcn.2024.101374)
Supplement: Supplementary file 1 — Supplementary material [file mmc1.docx]

**Supplemental Materials**

**Supplemental Materials Figure 1**

*Changes in the Number of Tracts from Prenatal to Postpartum as a Function of Length Threshold*

*
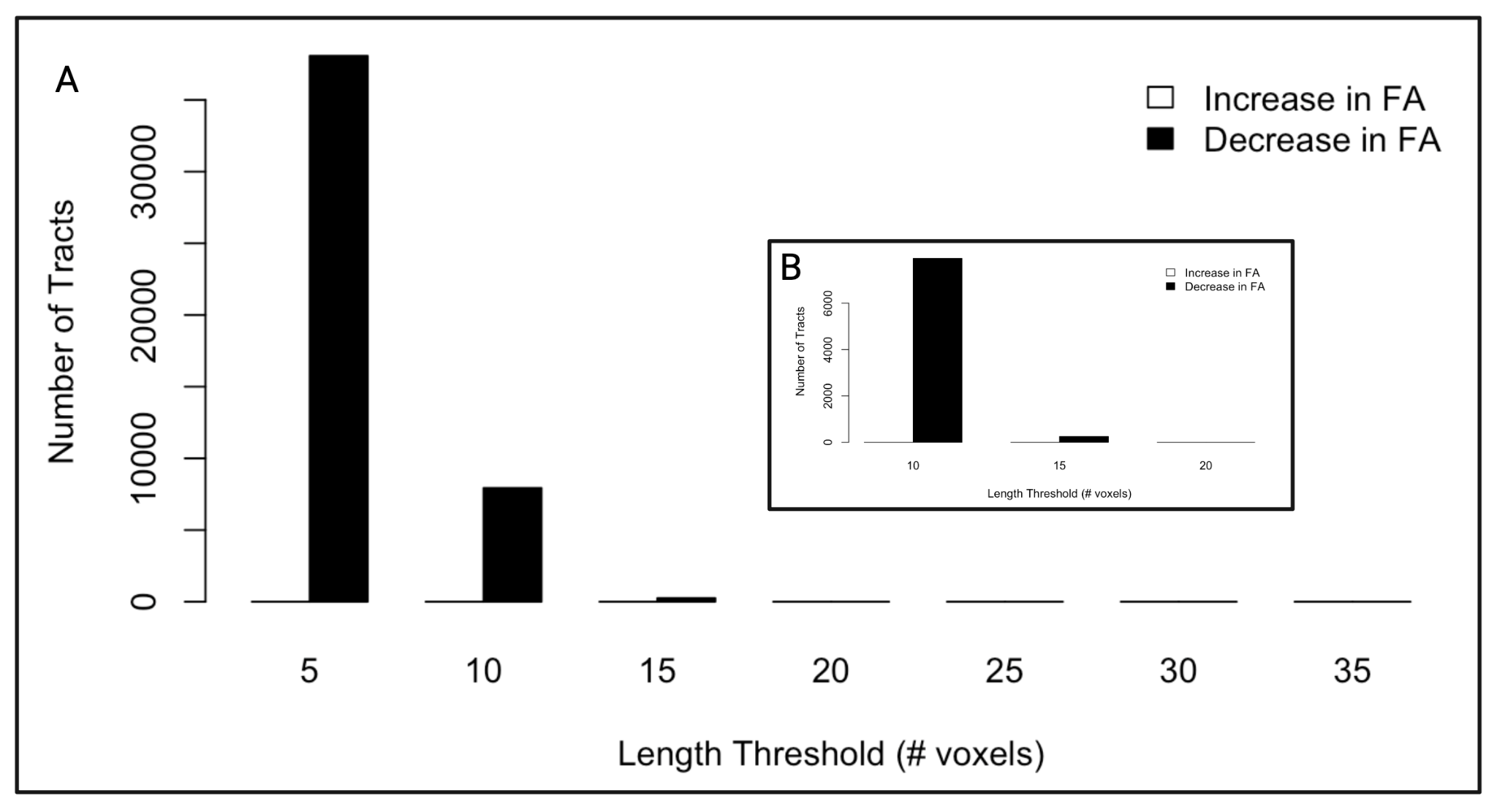
*

*Note.* (1A) Depicts the number of tracts that show a decrease in FA and increase in FA as a function of length thresholds between 0 and 35. (1B) Depicts Figure 1A in more detail; specifically, this figure depicts the number of tracts that show a decrease in FA and increase in FA as a function of length thresholds between 0 and 20.

**Supplemental Materials Figure 2**

**
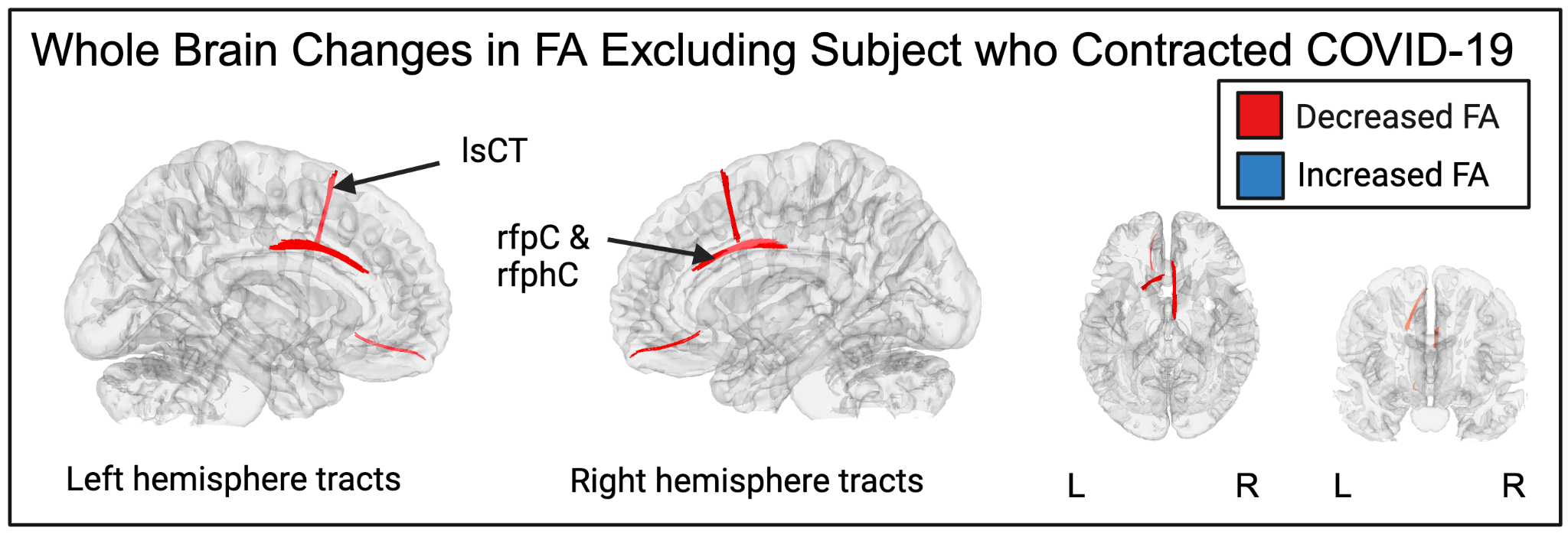
**

*Note.* Connectometry results of whole brain longitudinal changes in FA excluding subject who contracted COVID-19 (*n =* 29). Red represents tracts showing decreased FA. Three tracts are labeled. The cerebellum was excluded from the analysis and the seeding region was placed at the whole brain. T-score = 2, FDR < 0.05. FA = Fractional anisotropy, L = Left, R = Right, rfpC = Right frontal parietal cingulum, lsCT = Left superior corticostriatal tract, Right Frontal Parahippocampal Cingulum = rfphC.

**Supplemental Materials Figure 3**

**
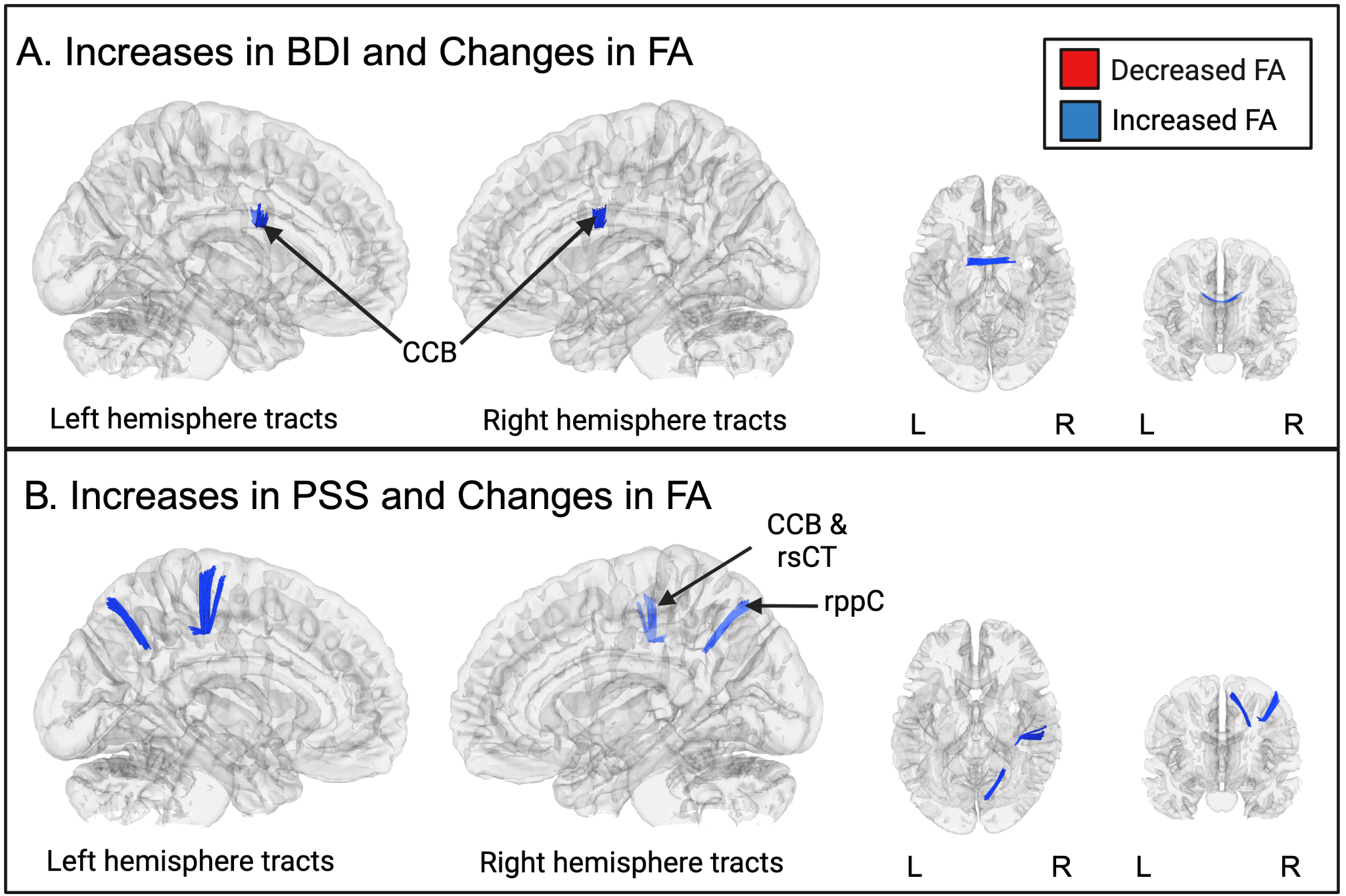
**

*Note.* Connectometry results of association between increases in BDI and longitudinal changes in white matter from prenatal to postpartum. Blue represents tracts showing increased FA. The cerebellum was excluded from the analysis and the seeding region was placed at the whole brain. T-score = 2., FDR < 0.05. (A) Association between higher BDI and longitudinal FA changes. One tract bundle labeled. (B) Association between increases in PSS and longitudinal changes. Three tracts labeled. FA = Fractional anisotropy, L = Left, R = Right, CCB = Corpus Callosum Body, rsCT = Right superior Cortico-striatal Tract, rppC = Right Parahippocampal Parietal Cingulum.

Supplemental Materials Table 2. *Effect Sizes for Variables in Significant Regression Models*

|  | Variables | | | | |
| --- | --- | --- | --- | --- | --- |
| Regression Models  (sample size) | Time | Participant’s Age at the Prenatal MRI Visit | Gestational Age of the Fetus at the Prenatal MRI Visit | Age of the Infant at the Postpartum MRI Visit | cohort |
| Whole Brain Decreased FA  (*n* = 30) | -.08 | .01 | -.01 | .05 | .01 |

*Note.* Time was operationalized as the number of months between scan 1 and scan 2.

Supplemental Materials Table 3. *Regions with Decreased Connectivity at Whole Brain Level*

| Bundle regions | Number of tracts | FDR |
| --- | --- | --- |
| Right Frontal Parietal Cingulum | 154 | 0.001 |
| Corpus Callosum Forceps Minor | 43 | 0.001 |
| Frontal Anterior Commissure | 15 | 0.001 |
| Left Anterior Thalamic Radiation | 13 | 0.001 |
| Right Parolfactory Cingulum | 10 | 0.001 |
| Left Anterior Cortico-striatal Tract | 7 | 0.001 |
| Left Superior cortico-striatal Tract | 5 | 0.001 |
| Right Frontal Parahippocampal Cingulum | 4 | 0.001 |
| Left Inferior Fronto Occipital Fasciculus | 1 | 0.001 |

Supplemental Materials Table 4. *Regions with Decreased Connectivity at Regional Level of Corpus Callosum*

| Bundle regions | Number of tracts | FDR |
| --- | --- | --- |
| Corpus Callosum Forceps Minor | 161 | 0.001 |
| Frontal Anterior Commissure | 55 | 0.001 |
| Left Anterior cortico-striatal Tract | 27 | 0.001 |
| Left Anterior Thalamic Radiation | 27 | 0.001 |
| Left Superior cortico-striatal Tract | 23 | 0.001 |
| Left Inferior Fronto Occipital Fasciculus | 14 | 0.001 |
| Right Arcuate Fasciculus | 10 | 0.001 |
| Corpus Callosum Body | 8 | 0.001 |
| Right Frontal Aslant Tract | 7 | 0.001 |
| Superior cortico-striatal Tract | 7 | 0.001 |
| Corpus Callosum Forceps Major | 7 | 0.001 |
| Left Uncinate Fasciculus | 5 | 0.001 |
| Left Non Decussating Dentatorubrothalamic Tract | 4 | 0.001 |
| RightUncinate Fasciculus | 2 | 0.001 |
| Superior Thalamic Radiation | 1 | 0.001 |
| Left Reticular Brainstem | 1 | 0.001 |

Supplemental Materials Table 5. *Regions with Changes in Connectivity at Whole Brain Level Associated with Higher Postpartum Depression Measured by the BDI-II*

| Bundle regions | Number of tracts | FDR |
| --- | --- | --- |
| *Decrease* |  |  |
| Left Cerebellum | 9 | 0.026 |
| Right Frontal Parietal Cingulum | 1 | 0.026 |
| Right Parahippocampal Parietal Cingulum | 1 | 0.026 |
| Right Cerebellum | 1 | 0.026 |
| *Increase* |  |  |
| Left Superior cortico-striatal Tract | 3 | 0.043 |
| Left Frontal Aslant Tract | 1 | 0.043 |
| Left Frontal Corticopontine Tract | 1 | 0.043 |

Supplemental Materials Table 6. *Regions with Reductions in Connectivity at Whole Brain Level Associated with Higher Postpartum Depression Measured by the EPDS*

| Bundle regions | Number of tracts | FDR |
| --- | --- | --- |
| Left Anterior Cortico-striatal Tract | 45 | 0.000 |
| Left Inferior Fronto Occipital Fasciculus | 30 | 0.000 |
| Left Anterior Thalamic Radiation | 11 | 0.000 |

Supplemental Materials Table 7. *Regions with Changes in Connectivity at Regional Corpus Callosum Level Associated with Higher Postpartum Depression Measured by the BDI-II*

| Bundle regions | Number of tracts | FDR |
| --- | --- | --- |
| *Decrease* |  |  |
| Corpus Callosum Forceps Major | 177 | 0.001 |
| Corpus Callosum Tapetum | 36 | 0.001 |
| Right Anterior cortico-striatal Tract | 24 | 0.001 |
| Right Parahippocampal Parietal Cingulum | 15 | 0.001 |
| Corpus Callosum Forceps Minor | 8 | 0.001 |
| Right Frontal Parietal Cingulum | 6 | 0.001 |
| Right Inferior Fronto Occipital Fasciculus | 4 | 0.001 |
| Left Frontal Parahippocampal Cingulum | 1 | 0.001 |
| Right Frontal Parahippocampal Cingulum | 1 | 0.001 |
| Right Uncinate Fasciculus | 1 | 0.001 |
| *Increase* |  |  |
| Left Frontal Aslant Tract | 23 | 0.002 |
| Left Superior cortico-striatal Tract | 22 | 0.002 |
| Left Frontal Corticopontine Tract | 4 | 0.002 |
| Left Non Decussating Dentatorubrothalamic Tract | 4 | 0.002 |

Supplemental Materials Table 8. *Regions with Changes in Connectivity at Regional Corpus Callosum Level Associated with Higher Postpartum Depression Measured by the EPDS*

| Bundle regions | Number of tracts | FDR |
| --- | --- | --- |
| *Decrease* |  |  |
| Left Anterior cortico-striatal Tract | 98 | 0.004 |
| Left Inferior Fronto Occipital Fasciculus | 82 | 0.004 |
| Corpus Callosum Forceps Major | 41 | 0.004 |
| Right Parahippocampal Parietal Cingulum | 22 | 0.004 |
| Left Anterior Thalamic Radiation | 22 | 0.004 |
| Right Superior cortico-striatal Tract | 17 | 0.004 |
| Corpus Callosum Body | 9 | 0.004 |
| Right Superior Thalamic Radiation | 5 | 0.004 |
| Corpus Callosum Forceps Minor | 5 | 0.004 |
| Left Posterior Thalamic Radiation | 3 | 0.004 |
| Left Brainstem Reticular Tract | 3 | 0.004 |
| Right Anterior cortico-striatal Tract | 2 | 0.004 |
| Right Frontal Parietal Cingulum | 1 | 0.004 |
| Left Posterior cortico-striatal Tract | 1 | 0.004 |
| *Increase* |  |  |
| Right Posterior cortico-striatal Tract | 22 | 0.014 |
| Right Superior Longitudinal Fasciculus 2 | 4 | 0.014 |

Supplemental Materials Table 9. *Regions with Changes in Connectivity*

| Bundle regions | Number of tracts | FDR |
| --- | --- | --- |
| Right Parolfactory Cingulum | 120 | 0.000 |
| Right Posterior cortico-striatal Tract | 58 | 0.000 |
| Left Inferior Fronto Occipital Fasciculus | 48 | 0.000 |
| Right Parietal Corticopontine Tract | 44 | 0.000 |
| Right Extreme Capsule | 39 | 0.000 |
| Right Corticospinal Tract | 27 | 0.000 |
| Right Non Decussating Dentatorubrothalamic Tract | 27 | 0.000 |
| Right Frontal Parietal Cingulum | 22 | 0.000 |
| Corpus Callosum Body | 22 | 0.000 |
| Left Reticular Tract | 20 | 0.000 |
| Right Parietal Aslant Tract | 19 | 0.000 |
| Corpus Callosum Forceps Minor | 19 | 0.000 |
| Left Frontal Aslant Tract | 17 | 0.000 |
| Left Posterior cortico-striatal Tract | 17 | 0.000 |
| Right Arcuate Fasciculus | 15 | 0.000 |
| Right Medial Lemniscus | 15 | 0.000 |
| Anterior Thalamic Radiation | 14 | 0.000 |
| Superior Thalamic Radiation | 12 | 0.000 |
| Left Formix | 12 | 0.000 |
| Superior Longitudinal Fasciculus 2 | 11 | 0.000 |
| Left Anterior Thalamic Radiation | 11 | 0.000 |
| Left Anterior cortico-striatal Tract | 10 | 0.000 |
| Anterior cortico-striatal Tract | 9 | 0.000 |
| Right Frontal Aslant Tract | 8 | 0.000 |
| Right Middle Longitudinal Fasciculus | 6 | 0.000 |
| Left Superior Longitudinal Fasciculus Left 2 | 4 | 0.000 |
| Left Non Decussating Dentatorubrothalamic Tract | 4 | 0.000 |
| Dentatorubrothalamic lr Tract | 2 | 0.000 |
| Superior Longitudinal Fasciculus 3 | 1 | 0.000 |
| Right Formix | 1 | 0.000 |
| Right Corticobulbar Tract | 1 | 0.000 |
| Right Frontal Corticopontine Tract | 1 | 0.000 |

Supplemental Materials Table 10. *Regions with Decreased Connectivity at Whole Brain Level Excluding Subject who Contracted COVID*

| Bundle regions | Number of tracts | FDR |
| --- | --- | --- |
| Right Frontal Parietal Cingulum | 74 | 0.005 |
| Left Superior Cortico-striatal Tract | 30 | 0.005 |
| Right Frontal Parahippocampal Cingulum | 6 | 0.005 |
| Corpus Callosum Forceps Minor | 4 | 0.005 |
| Left Non Decussating Dentatorubrothalamic Tract | 2 | 0.005 |
| Right Parolfactory Cingulum | 1 | 0.005 |
|  |  |  |

Supplemental Materials Table 11. *Regions with Changes in Connectivity at Whole Brain Level Associated with Increased Prenatal to Postpartum Depression Measured by the BDI-II*

| Bundle regions | Number of tracts | FDR |
| --- | --- | --- |
| *Increase* |  |  |
| Corpus Callosum Body | 49 | 0.000 |

Supplemental Materials Table 12. *Regions with Changes in Connectivity at Regional Corpus Callosum Level Associated with Increased Prenatal to Postpartum Depression Measured by the PSS*

| Bundle regions | Number of tracts | FDR |
| --- | --- | --- |
| *Increase* |  |  |
| Right Parahippocampal Parietal Cingulum | 31 | 0.003 |
| Corpus Callosum Body | 20 | 0.003 |
| Right Superior Cortico-striatal Tract | 19 | 0.003 |
| Right Superior Thalamic Radiation | 9 | 0.003 |
| Righ Frontal Parietal Cingulum | 4 | 0.003 |
